# Sunglasses to hide behind may also prevent melanoma of the eyes

**DOI:** 10.1038/s41416-021-01343-8

**Published:** 2021-04-06

**Authors:** Nathalie Dhomen, Piyushkumar A. Mundra, Richard Marais

**Affiliations:** Molecular Oncology Group, Cancer Research UK Manchester Institute, The University of Manchester, Alderley Park, SK10 4TG UK

**Keywords:** Melanoma, Melanoma

## Abstract

In 1967, Sandy Posey pronounced that sunglasses are essential beachwear (https://www.youtube.com/watch?v=4HVBEb-GA1Y). Now, whole-genome sequencing reveals that ultraviolet radiation (UVR) can contribute to melanomas in the iris and conjunctiva, data that provide a molecular explanation for why it is important to protect our eyes from exposure to UVR.

## Main

**Fig. 1:**
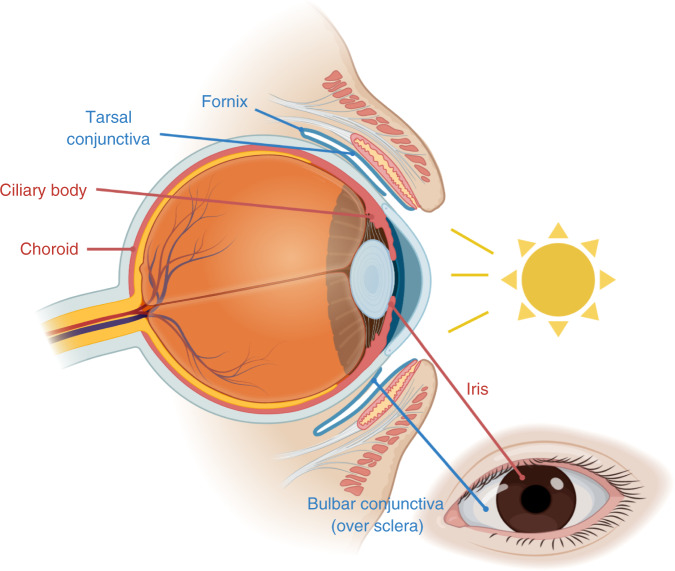
Areas of the eye in which melanoma arises. Sagittal cross-section and frontal view of the eye indicating the location of the components of the uveal tract (choroid, ciliary body and iris; red) and the conjunctiva (tarsal conjunctiva, fornix and bulbar conjunctiva; blue). The iris and bulbar conjunctiva are the sun-exposed areas of the uveal tract and conjunctiva respectively. Created with BioRender.com.

The American Joint Committee on Cancer (AJCC) staging system emphasises body site as important for melanoma diagnosis,^1^ because site predicts broad clinical characteristics and therefrom therapeutic options. Common cutaneous melanomas (~90% of cases) arise in the skin. More rarely, melanomas arise in the mucosa (~1–2% of cases) of, for example, the gastrointestinal and genitourinary tracts, and in the eyes (~5% of cases), either within the uveal tract (choroid, iris and ciliary body) or in the conjunctiva (the mucosa covering the sclera and lining the insides of the eyelids) (see Fig. 1). Of particular concern, these rare melanoma subtypes have limited treatment options.

Many epidemiological and experimental studies link common cutaneous melanoma of non-glabrous skin to exposure to ultraviolet radiation (UVR) and their genomes consequently show evidence of direct UVR-induced DNA damage, presenting with high tumour mutation burdens (TMBs), large numbers of C>T transitions and predominance of a mutational process known as single base substitution signature 7 (SBS7).^2^ Rare melanomas by contrast generally have low TMB and low proportions of C>T transitions and SBS7, but instead have large numbers of genome structural variations (SVs) with characteristic chromosomal gains and losses. The different melanoma subtypes are also driven by distinct oncogenes. Cutaneous melanomas are typically driven by *BRAF*, *NRAS* and *NF1*, with secondary mutations in *hTERT* and *TP53*. Mucosal melanomas are driven by *BRAF* and *NRAS* but at a lower frequency than cutaneous melanoma, and *KIT* is also a frequent driver in this disease. Lastly, uveal melanomas are driven by *GNAQ*, *GNA11* and *CYSLTR2*, with secondary mutations in *BAP1*, *SF3B1* and *EIF1AX*.^3–6^

Thus, different melanoma subtypes have distinct aetiologies, but recent genomic evidence suggests that these distinctions are not so clear-cut. In 2018, we reported that ~15% of common cutaneous melanomas do not appear to be UVR-driven,^2^ causing us to question by analogy if UVR is involved in some rare melanomas. In this context, the eye is particularly intriguing, because although the uveal tract is largely sun-protected, the iris is sun-exposed, and the conjunctiva is unusual because it is a partially sun-exposed mucosal membrane (Fig. 1). Sun exposure causes conditions such as photokeratitis, pinguecula, pterygium, cataracts and macular degeneration, and three recent papers now establish that it also contributes to melanoma of the eye.

The first paper, from Nicholas Hayward and colleagues^7^ reported whole-genome sequencing (WGS) of 103 uveal melanomas. As expected, these melanomas generally presented low TMBs, but eight were from the iris and they all presented high TMB and SBS7 predominance. Intriguingly, despite this clear evidence of UVR-induced DNA damage, the iris melanomas were still driven by mutant *GNAQ*, *GNA11* or *CYSLTR2*, and six samples also had mutations in *BAP1*, *EIF1AX* or *SF3B1*.

The other two papers reported the genomic landscapes of conjunctival melanomas. Following a brief earlier report,^8^ Carlo Rivolta and colleagues^9^ have now analysed WGS on two conjunctival melanomas and whole-exome sequencing on a further 12. They reported high proportions of copy number variants, but also high TMBs and high proportions of C>T transitions. *BRAF*, *NRAS, HRAS* or *NF1* were mutated in 12 lesions, *hTERT* was mutated in nine, and *TP53* was also mutated in nine. Notably, SBS7 was predominant, but the association between SBS7 and presumed sun exposure was weak.

In the third paper, we reported WGS of ten conjunctival melanomas compared with eight other mucosal melanomas and 54 cutaneous melanomas.^10^ The conjunctival melanomas had genome SVs that were similar to the other mucosal melanomas, but nine also had high TMBs, high proportions of C>T transitions and SBS7 predominance. Six of the nine had *BRAF*, *NRAS* and *NF1* mutations, four had *hTERT* mutations, two had *TP53* mutation, and eight had mutations in 1 to 11 genes recurrently mutated in common cutaneous melanoma. Notably, five of the SBS7-dominant lesions were from the sun-exposed bulbar conjunctiva and the non-SBS7 lesion was from the largely sun-protected fornix, but one SBS7-dominant lesion spanned sun-exposed and sun-protected sites and three were from the largely sun-protected tarsal conjunctiva (Fig. 1). In our validation cohort, UVR involvement was evident in some lip and gingiva mucosal melanomas, which can be sun-exposed, but also in the nasal cavity and oropharynx mucosal melanomas, which are sun-protected. Thus, our data also showed a weak association between UVR signatures and presumed sun exposure sites.

There is interesting biology to explore, but these data show that UVR plays a role in uveal melanomas in the iris, most conjunctival melanomas and some mucosal melanomas at other sun-exposed sites. Curiously, despite clear UVR signatures, the iris melanomas were still driven by uveal melanoma oncogenes and the conjunctival melanomas still had the genomic SVs characteristic of mucosal melanoma. It is unclear what drives mutagenesis in uveal and mucosal melanomas, but the persistence of baseline genomic features in the UVR-driven lesions suggests that they are peculiar to the different microenvironments in which melanocytes reside, and we posit that UVR accelerates melanomagenesis at various sites by imposing additional events over the existing site-specific processes. That 15% of skin melanomas do not appear to be UVR-driven^2^ suggests that the skin microenvironment also has an underlying process that can be accelerated by UVR.

These new findings have two important implications. First, they show that UVR can damage melanocytes in the eye to drive melanomagenesis, emphasising the importance of public health campaigns advocating sunglasses to protect the eyes from UVR. This is especially important in strong sunlight, such as on the beach or during activities including driving, boating and skiing. Second, the genomes of UVR-driven rare melanomas are remarkably similar to those of UVR-driven cutaneous melanomas for which targeted therapies and immunotherapies are approved, and we advocate therefore that UVR-driven rare melanomas should be considered for these therapies. Accepting the eye as an immune-privileged site, we nevertheless advocate immunotherapies for UVR-driven iris melanoma, particularly in the metastatic setting. For UVR-driven conjunctival and other mucosal melanomas, we again advocate immunotherapies, or if tractable *BRAF* mutations are present, BRAF/MEK inhibitors. As described above, the site of a melanoma does not immediately implicate UVR. Therefore, to facilitate administration of these drugs, we propose a simple assay based on targeted sequencing of only ten genes (*LRP1B*, *GPR98*, *XIRP2*, *PKHD1L1*, *USH2A*, *DNAH9*, *PCDH15*, *DNAH10*, *TP53* and *PCDHAC1*) that can reveal UVR involvement with reasonable certainty, and which is more convenient and cost-effective than WGS.^2,10^

## Data Availability

Not applicable.
